# Prognostic factors for tube feeding in type I SMA patients treated with disease-modifying therapies: a cohort study

**DOI:** 10.1007/s00431-024-05735-9

**Published:** 2024-08-29

**Authors:** Marika Pane, Giulia Stanca, Giorgia Coratti, Adele D’ Amico, Valeria Ada Sansone, Beatrice Berti, Lavinia Fanelli, Emilio Albamonte, Carolina Ausili Cefaro, Antonella Cerchiari, Michela Catteruccia, Roberto De Sanctis, Daniela Leone, Concetta Palermo, Bianca Buchignani, Roberta Onesimo, Eliza Maria Kuczynska, Michele Tosi, Maria Carmela Pera, Chiara Bravetti, Francesco Danilo Tiziano, Enrico Bertini, Eugenio Mercuri

**Affiliations:** 1 Pediatric Neurology, Università Cattolica del Sacro Cuore, Rome, Italy; 2 Centro Clinico Nemo, Neuropsichiatria Infantile, Fondazione Policlinico Universitario Agostino Gemelli IRCCS, Rome, Italy; 3 Unit of Muscular and Neurodegenerative Disorders, Bambino Gesù Children’s Hospital, IRCCS, Rome, Italy; 4 The NEMO Center in Milan, Neurorehabilitation Unit, University of Milan, ASST Niguarda Hospital, Milan, Italy; 5 Speech Language Pathology Unit, Fondazione Policlinico Universitario Agostino Gemelli IRCCS, Rome, Lazio Italy; 6 Feeding and Swallowing Services Unit, Bambino Gesù Children’s Hospital IRCCS, Rome, Italy; 7 Department of Translational Research and of New Surgical and Medical Technologies, University of Pisa, Pisa, Italy; 8 Center for Rare Diseases and Birth Defects, Department of Woman and Child Health and Public Health, Fondazione Policlinico Universitario A. Gemelli IRCCS, Rome, Italy; 9 Department of Life Sciences and Public Health, Section of Genomic Medicine, Università Cattolica del Sacro Cuore, Rome, Italy

**Keywords:** Spinal muscular atrophy, Swallowing, Oro-bulbar, Disease-modifying therapies

## Abstract

The aim of this study was to assess the need for tube feeding in a cohort of treated infants with type I SMA and to identify predictive factors. All patients were classified at baseline, when treatment started, and at follow-up according to their functional level and the need for tube feeding. Fisher’s exact test was used to examine the associations between the outcome at the last follow-up and SMA type, SMN2 copy number, and baseline nutritional status. ANOVA was performed to compare CHOP INTEND scores and age at treatment initiation with outcomes. The cohort includes 75 type I SMA infants treated between 0.1 and 5 years of age. At the last follow-up, 34 had no need for tube feeding, 9 had tube feeding but were also able to be fed by mouth, and 32 had tube feeding and were unable to be fed by mouth. Thirty of the 41 infants with tube feeding at follow-up already had feeding difficulties when treatment was started. The need for tube feeding at follow-up was associated with the level of feeding involvement at baseline and with CHOP INTEND scores [*p* < 0.001] but not with SMN2 copy number, SMA type 1 subtypes or age at treatment. The results of this study suggest that the need for tube feeding is not frequent in treated infants with type I SMA and, when occurring, can be predicted by the level of feeding involvement and low CHOP INTEND scores at baseline.

**Table:** 

What is Known: • *The advent of disease-modifying therapies is increasingly changing the approach to swallowing and nutritional management in type I SMA.* • *Clinical trials and real-world data using all three disease-modifying therapies report a rather wide variability of feeding outcome and need for tube feeding that is often related to different cohorts that makes comparison between studies very difficult.*
What is New: • *The real-world findings of this study, including all the children treated since treatments became available, confirmed that the need for tube feeding is not an invariable finding.* • *The level of feeding involvement at baseline appears to be a reliable prognostic indicator of bulbar outcome.* • *The results highlight the need for interventional studies with structured Speech and Language Therapist protocols that will help to better understand the extent to which bulbar function can be maintained or regained even in children requiring tube feeding.*

**Supplementary Information:**

The online version contains supplementary material available at 10.1007/s00431-024-05735-9.

## Introduction

Spinal muscular atrophy (SMA) is caused by pathogenic variants in the survival motor neuron 1 (*SMN1*) gene leading to loss of motoneurons in the anterior horns and subsequent muscle atrophy and weakness [1]. In the pre-treatment era type 1 SMA, the most severe form with onset before the age of 6 months was classically characterized by inability to achieve the ability to sit unsupported and by progressive weakness. Progressive feeding involvement was an invariable finding, generally requiring feeding support by 12 months of age [2–4]. The advent of disease-modifying therapies has dramatically changed not only survival but also the possibility to develop severe clinical signs, including swallowing impairment [5–7]. Both clinical trials and real-world data show that the risk of developing severe bulbar impairment is much lower than in the past and becomes progressively lower if infants receive early treatment soon after diagnosis [5–9] or, if identified through neonatal screening, before the onset of clinical signs of SMA [10–12].

Until recently, swallowing abilities have not been systematically explored in treated SMA patients. Even the pivotal clinical trials did not systematically assess these aspects [5–8], with the exception of recent gene therapy studies in which the assessment was based on the clinician perspective rather than on structured assessments [9, 10]. More attention has recently been devoted to the development of appropriate tools specifically designed for assessing bulbar function in SMA and with increased attention to systematically capture swallowing abilities over time [13–18].

Using these tools, it has been possible to establish that swallowing abilities are overall more preserved in treated infants, even though there is some variability and a number of children still require tube feeding [5–8, 17]. Even when tube feeding is required, infants may improve or even regain the ability to eat by mouth after Percutaneous Endoscopic Gastrostomy (PEG) insertion, something which had previously not been observed in untreated patients [19].

The aim of this longitudinal prospective study was to assess the need for tube feeding in a cohort of infants with type I SMA treated at different ages. Additionally, we aimed to assess if there are factors that may predict feeding outcomes at the time treatment is started. Limited data are available on this topic and this could be important for the management of these patients.

## Methods

As part of the activities of a nation-wide registry, data were prospectively collected using a structured electronic Case Report Form (eCRF) [20]. All individuals who had a confirmed genetic diagnosis of SMA and a clinical diagnosis of type I SMA were considered for inclusion in the study, no exclusion criteria were applied to the cohort. This prospective study was approved by the institutional Ethics Committee and all parents signed consent forms.

Infants were classified according to the Dubowitz’s decimal classification [21]: 1.1, characterized by early, severely reduced mobility, respiratory and bulbar difficulties; 1.5, the most common phenotype, with inability to raise the legs against gravity but no feeding or respiratory difficulties at diagnosis; 1.9, the mildest phenotypes, infants achieve head control [21, 22]. All had been prospectively followed since diagnosis even if therapy in some cases was started at a later age when the drugs became commercially available.

### Baseline

At baseline (at the time treatment was started), all patients were assessed using a clinical assessment of swallowing abilities and, if swallowing difficulties were suspected, also with videofluoroscopy. Based on the assessment at baseline, the cohort was subdivided into three subgroups according to the same criteria used in a previous study [19]: (a) no obvious clinical signs of feeding involvement and no need for tube feeding; (b) tube feeding or evidence of swallowing difficulties requiring tube feeding; (c) tube feeding and tracheostomy.

The cohort was also classified using the Oral Swallowing Ability Tool (OrSAT), a new tool specifically designed to assess swallowing in weak SMA infants [23]. In this study, we only used the part of the OrSAT scale that allows to identify four levels of impairment as, unlike the OrSAT checklist that so far been validated until the age of 24 months [23], this can be applied independently of the age. The levels include *severe impairment* (level 1): unable to swallow by mouth, tube needed; *moderate impairment* (level 2): able to swallow some food consistencies safely but need for oral supplements or tube feeding; *mild impairment* (level 3): safe swallowing but requires compensatory strategies or other intervention; and *no impairment* (level 4) safe and efficient swallowing for all consistencies.

All children were also assessed using the Children’s Hospital of Philadelphia Infant Test of Neuromuscular Disorders (CHOP INTEND), a motor functional scale specifically designed for type I SMA infants [24].

### Follow-up

All patients were followed regularly at least every 6 months and information on nutritional changes were recorded. As part of the policy of all 3 centers participating in the study, all patients with known swallowing difficulties, including those who had tube feeding, were part of Speech and Language Therapist (SALT) rehabilitation programs. The outcome at the last visit was subdivided into three subgroups: (1) no need for tube feeding; (2) tube feeding but associated with some ability to eat by mouth; (3) tube feeding with inability to eat anything by mouth.

### Statistical analysis

The statistical analysis was conducted using R version 4.1.1. Descriptive statistical techniques were employed to outline the clinical and demographic features of the sample. The cohort was stratified into SMA I subtypes based on criteria aligned with the Dubowitz classification [21] by baseline OrSAT levels (severe, moderate, mild impairment, or no impairment) and by baseline nutritional status (categorized as oral fed or tube fed). No missing data were found in the registry dataset.

Quantitative variables were summarized using minimum, maximum, median, mean, and standard deviation. The distribution of continuous variables was evaluated using the Shapiro–Wilk test. To explore the associations between the outcome at the last follow-up (categorized as oral fed, tube fed, or both oral and tube fed) and SMA type, OrSAT levels, SMN2 copy number and nutritional status at baseline, Fisher’s exact test was employed.

In addition, an analysis of variance (ANOVA) was performed to compare CHOP Intend scores at treatment initiation with outcomes, as well as age at treatment initiation with outcomes. A *p*-value of less than 0.05 was considered statistically significant.

## Results

The cohort includes 75 type I SMA patients (38 females and 37 males) with pathogenic variants of *SMN1*, seen in the participating centers between January 2020 and November 2022, who had at least 12-month follow-up. They were all treated with the available disease-modifying therapies soon after diagnosis or, in the older patients, when the treatment became available.

Thirteen of the 75 patients were classified as type 1.1, 41 as type 1.5 and the remaining 21 as 1.9. The age when treatment was started ranged between 0.1 and 5 years (mean 1.3). Follow-up after treatment ranged between 1 and 7.7 years. One patient had one SMN2 copy, 63 had 2 copies, and 11 had 3 copies.

Sixty-nine of 75 patients were treated with Nusinersen, 18/69 switched to Onasemnogene Abeparvovec (OA), and another 5/69 to Risdiplam. Another 6 patients were treated with Onasemnogene Abeparvovec only.

### Baseline

At the time treatment was started 45/75 infants had no obvious clinical signs of feeding involvement and no need for tube feeding. The remaining 30 had clinical and/or videofluoroscopy evidence of swallowing difficulties and tube feeding had already been inserted or was inserted at the time when they came to observation to start treatment. Sixteen of the 30 also had shown severe respiratory insufficiency requiring tracheostomy before treatment was started.

OrSAT levels at baseline were available in 69 out of the 75 patients and this showed no impairment in 19, mild or moderate impairment in 30, and severe impairment in 20 patients.

### Outcome

At the last follow-up, 34 of the 75 had no need for tube feeding. Nine had tube feeding but were also able to be fed by mouth and 32 had tube feeding and were unable to be fed by mouth. Table 1 shows the characteristics of the cohort at baseline.

**Table 1:** Baseline characteristics of the cohort

	Whole cohort (*N* = 75)	No need for tube feeding (*N* = 34)	Tube feeding and oral (*N* = 9)	Tube feeding without oral (*N* = 32)
Age				
Mean (SD)	1.34 (1.19)	1.18 (0.98)	1.08(1.14)	1.58 (1.39)
Median [range]	0.81 [0.06–4.98]	0.83 [0.06–4.98]	0.63 [0.23–3.73]	0.92 [0.24–4.78]
Gender				
F, *n* (%)	38 (51)	16 (47)	5 (56)	17 (53)
M, *n* (%)	37 (49)	18 (53)	4 (44)	15 (47)
SMN2 copies				
1, *n* (%)	1 (1)	0 (0)	0 (0)	1 (3)
2, *n* (%)	63 (84)	27 (79)	8 (89)	28 (88)
3, *n* (%)	11 (15)	7 (21)	1 (11)	3 (9)
SMA subtype				
1.1, *n* (%)	13 (17)	4 (12)	2 (22)	7 (22)
1.5, *n* (%)	41 (55)	16 (47)	6 (67)	19 (59)
1.9, *n* (%)	21 (28)	14 (41)	1 (11)	6 (19)
Tube feeding				
Yes, *n* (%)	30 (40)	0 (0)	4 (44)	26 (81)
No, *n* (%)	45 (60)	34 (100)	5 (56)	6 (19)
Tracheostomy				
Yes, *n* (%)	16 (21)	0 (0)	0 (0)	16 (50)
No, *n* (%)	59 (79)	34 (100)	9 (100)	16 (50)
Treatment				
Nusinersen, *n* (%)	69 (92)	29 (85)	9 (100)	31 (97)
OA, *n* (%)	6 (8)	5 (15)	0 (0)	1 (3)
Risdiplam, *n* (%)	0 (0)	0 (0)	0 (0)	0 (0)
Switch				
Nus. + OA, *n* (%)	18 (24)	14 (41)	2 (22)	2 (6)
Nus. + Risd, *n* (%)	5 (7)	0 (0)	0 (0)	5 (16)

**Fig. 1:**
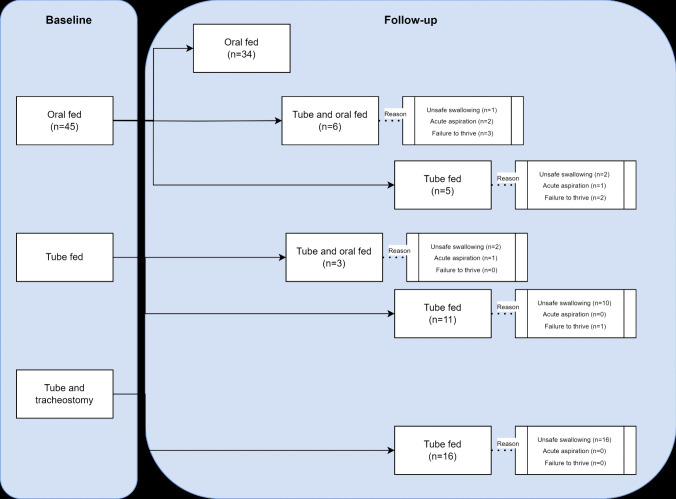
Reasons for inserting PEG in the cohort subdivided according to baseline and follow-up findings

Details of the reasons for inserting PEG in the cohort subdivided according to baseline and follow-up findings are shown in Fig. 1.

### Feeding outcome and gender

The association between gender and the three outcome groups was not significant (*p* = 1.0).

### Feeding outcome and SMA I subtypes

**Fig. 2:**
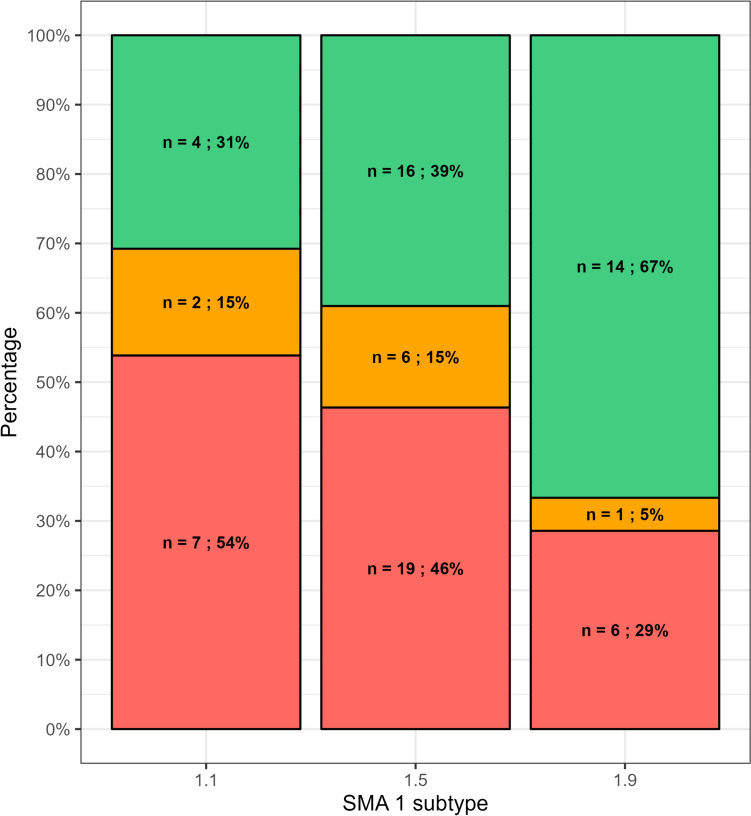
Association between SMA I subtypes and the three outcome groups. Key to figure: red = tube fed; orange = tube and oral fed; green = oral fed

The association between SMA I subtypes and the three outcome groups (oral fed, tube fed, tube and oral fed) was not significant (*p* = 0.21) (Fig. 2).

### Feeding outcome and SMN2 copies

**Fig. 3:**
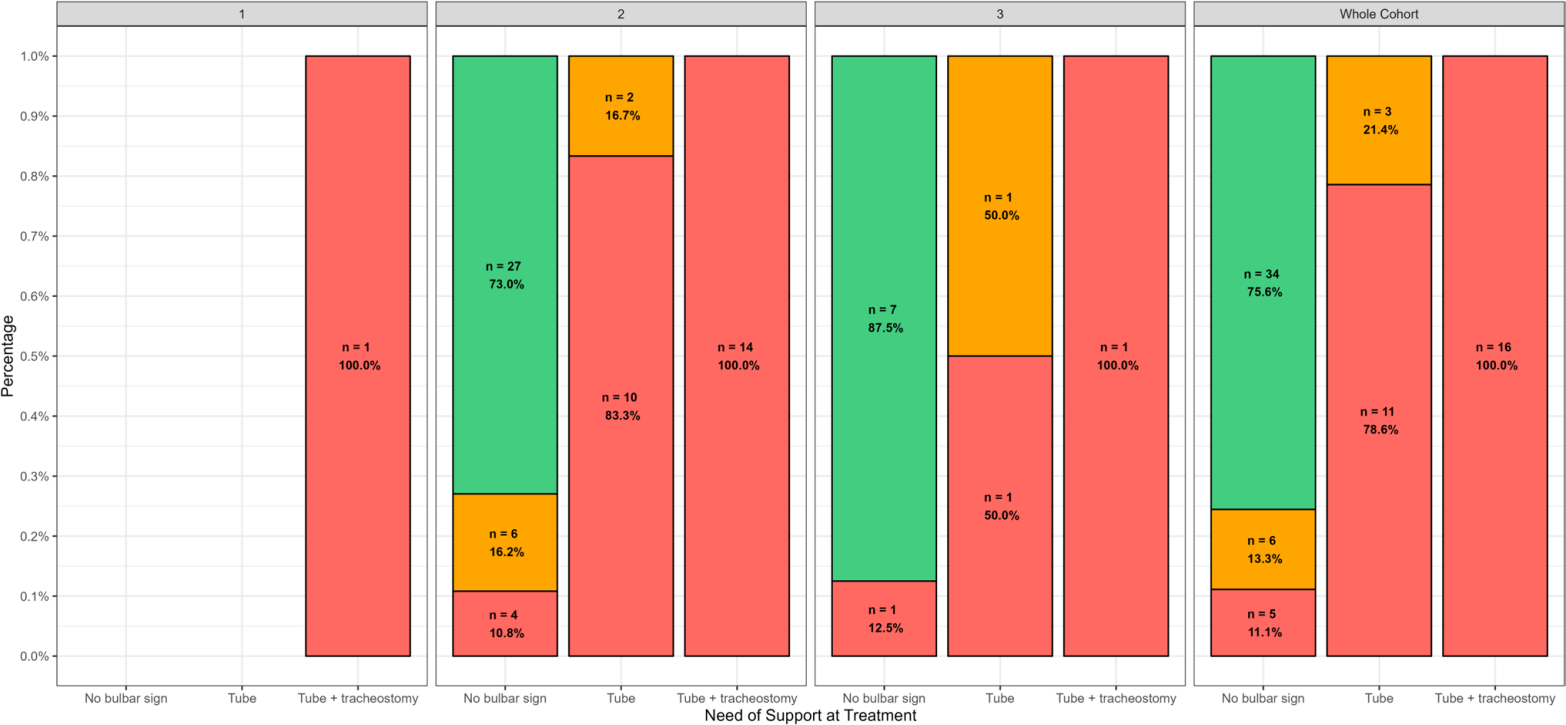
SMN2 copies and the three outcome groups subdivided by need of support at treatment. Key to figure: red = tube fed; orange = tube and oral fed; green = oral fed

The association between SMN2 copies and the three outcome groups (oral fed, tube fed, tube and oral fed) subdivided by SMN2 copy number was not significant (*p* = 0.53). Figure 3 show patients’ outcome subdivided by need of support at treatment and SMN2 copies.

### Feeding outcome and age at treatment

There was no significant difference in age at treatment among the three outcome groups (oral fed, tube fed, tube and oral fed), *F*(2, 22.4) = 1.05, *p* = 0.37.

### Feeding outcome and overall need for support at treatment initiation

There was a significant association between the overall need for support at treatment initiation (no need for tube support, need for tube support, need for tube support and tracheostomy) and the three outcome groups (oral fed, tube fed, tube and oral fed) (*p* < 0.001).

### Feeding outcome and OrSAT levels at the time of treatment

**Fig. 4:**
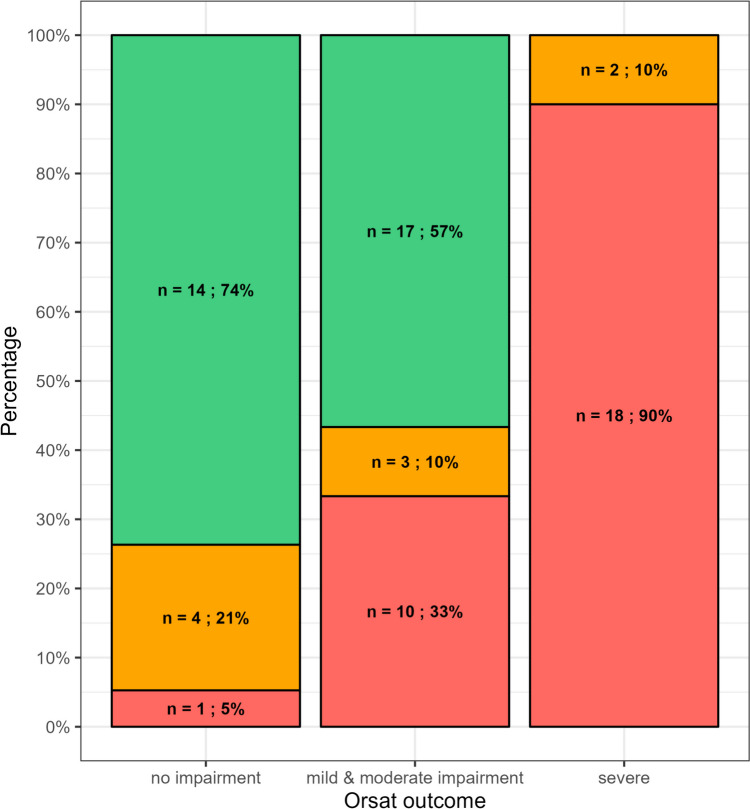
Association between OrSAT levels at treatment initiation and the three outcome groups. Key to figure: red = tube fed; orange = tube and oral fed; green = oral fed

There was a significant association (*p* < 0.001) between OrSAT levels at treatment initiation (severe, moderate, mild impairment, or no impairment) and the three outcome groups (oral fed, tube fed, tube and oral fed) (Fig. 4).

### Feeding outcome and CHOP INTEND at the time of treatment initiation

**Fig. 5:**
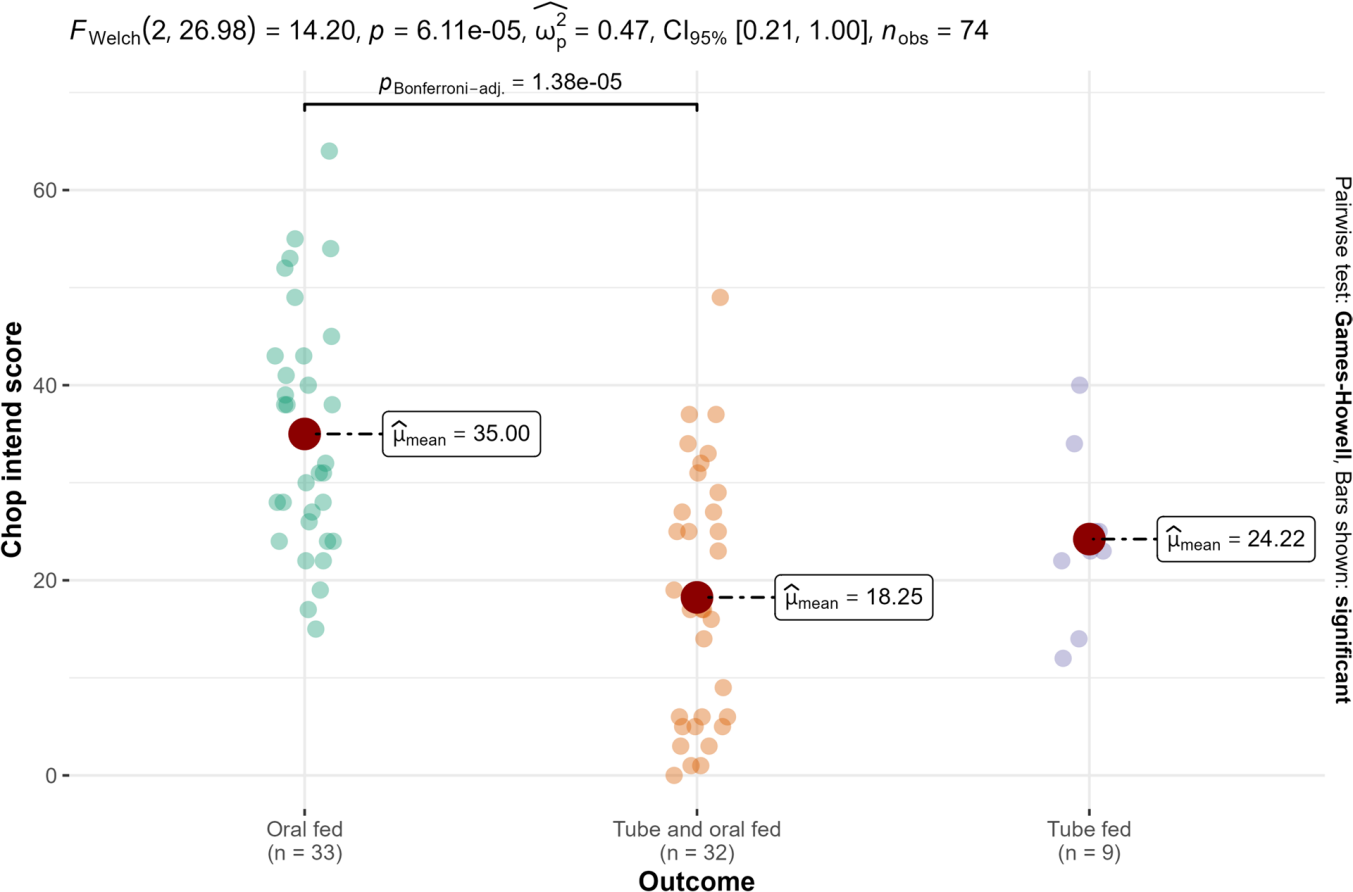
CHOP INTEND at treatment initiation and three outcome groups

There was a significant difference in CHOP INTEND at treatment initiation among the three outcome groups (oral fed, tube fed, tube and oral fed), *F*(2, 27) = 14.20 (*p* < 0.001) (Fig. 5).

### Feeding outcome and type of treatment

As most infants (92%) had Nusinersen as the first treatment, and the number of those who switched or who had Onasemnogene Abeparvovec was small, we were unable to perform a meaningful statistical analysis to ascertain possible differences among treatments. Figure 1S provides individual details of the type of treatment and of the age when this was started.

## Discussion

In this study, we aimed to assess the need for tube feeding in a cohort of type I SMA infants treated with the available therapies and to establish if any of the information available at the time when treatment was started could predict feeding outcome.

This real-world data, including all the children treated since treatments became available, showed that at the last follow-up, nearly half of treated infants had no need for tube feeding. Over 70% of those who were tube fed at follow-up were already tube fed at the time when treatment started. If we only consider the 45 patients who had no obvious clinical signs of feeding difficulties when treatment was started, the percentage of those not requiring tube feeding at follow-up was nearly 80%, with only 11 patients requiring tube feeding after treatment (14.6% of the whole cohort).

Bulbar function at the time of treatment initiation appeared to be a strong predictor of need for tube feeding at follow-up, as also shown by the significant association between need for tube feeding and baseline OrSAT levels. The need for tube feeding was also associated with the overall severity of motor function, with higher risk of tube feeding in infants with lower CHOP INTEND scores. These findings are concordant with a recent study also highlighting the strong predictive value of early neurological signs to identify infants with more severe outcome and poor prognosis [25].

Infants with 2 SMN2 copies and with early onset (1.1 SMA) were at higher risk of need for tube feeding, but the association was not significant, as the outcome in infants with 2 SMN2 copies or 1.1 SMA was variable. Similarly, the overall association between age when treatment was started, or gender and feeding outcome was also not significant.

These findings also provide information on the changes in the observed phenotypes and in management. The need to harmonize SALT protocols was recently highlighted in a report of a meeting between experts [26], suggesting that more proactive approaches in treated patients, even in the presence of tube feeding. A number of infants in this cohort were able to maintain or regain the ability to eat by mouth after tube feeding was inserted. This occurred in over half of the infants (6/11) who required tube feeding after pharmacological treatment and in approximately 20% (3/14) of those who were already tube fed (with no tracheostomy) at the time when pharmacological treatment was started. These findings, obtained as part of regular SALT sessions with careful monitoring of the reintroduction of food in a protected setting expand the possibility to distinguish, among the patients who require tube feeding, a subset with a more favorable outcome. The ability to eat something by mouth is not only important as a sign of lesser bulbar involvement but is also very relevant for quality of life, participation to family meals, etc. These findings also highlight that other factors, such as the reason for tube feeding insertion, may contribute to better explain the variable outcome. In the past, in untreated patients swallowing difficulties were nearly invariable and always progressive. Because of this, tube feeding was promptly suggested at the first signs of feeding difficulties to avoid aspirations and was often recommended to be inserted even before the onset of swallowing difficulties [27]. The care recommendations redacted before the therapies became available suggested tube feeding not only for obvious signs or swallowing difficulties but also for failure to thrive or fatigue during meals or following an acute episode of aspiration pneumonia, generally occurring during an infection. Even if limited by small numbers and by the fact that videofluoroscopy was not systematically available, the results obtained show that the possibility to maintain or regain the ability to eat by mouth was higher in the infants with temporary acute events or failure to thrive that had possibly not permanently affected the ability to swallow. These findings also highlight the need to rediscuss care recommendations. The increasing evidence of efficacy of the new treatments on bulbar function, together with a more careful and systematic approach for monitoring feeding and swallowing abilities, is already increasingly resulting in a less interventional approach in cases with single acute episodes of unsafe swallowing occurring during an infection, with insertion of tube feeding only if signs were persisting after recovery of the acute event.

This study has limitations. First, most infants in this cohort were initially treated with Nusinersen, the first available drug, with a relatively small number of patients switching to gene therapy or starting other drugs as first treatment, this impeding any analysis to establish differences among the available treatments. Another limitation is that videofluoroscopy was only performed in a limited number of cases with clinical signs of possible feeding involvement. This reduces the possibility to establish if the relatively small proportion with no obvious clinical signs at baseline who later require tube feeding may have shown minimal signs on videofluoroscopy that would have allowed early identification of their difficulties.

Even with these limitations, this study provides useful information on the possibility to early identify children at higher risk for tube feeding. The level of feeding involvement at baseline appears to be a reliable prognostic indicator of feeding outcome confirming the results already reported in previous studies [16, 19, 28]. Other factors, such as a reduced SMN2 copy number, SMA type 1.1 subtype or treatment started at a later age, are also related to an increased risk for tube feeding but their prognostic value is partly limited by the relatively high variability observed for each of them. Further studies in larger cohorts treated with different drugs and with the aid of videofluoroscopy will help to better identify children at higher risk of tube feeding and to establish possible differences among the available treatments.

Finally, these results, even if limited by the small number of cases and the lack of a harmonized SALT protocol, highlight the need for interventional studies with structured SALT protocols that will help to better understand the extent to which feeding function can be maintained or regained even in children requiring tube feeding.

## Supplementary Information

Below is the link to the electronic supplementary material.

Supplementary file1 Figure 1S. Individual details of the type of treatment and of the age when this was started. Key to the figure. Asterisk: number of SMN2 copies; red circle: insertion of PEG; blue arrow: restarted to eat by mouth; black cross: insertion of tracheostomy. The green lines indicate treatment with onasemnogene abeparvovec, the orange lines indicate treatment with Nusinersen; the yellow lines indicate treatment with Risdiplam and the grey ones indicate the time without any treatment. (PNG 145 kb)

High resolution image (TIF 412 kb)

## Data Availability

Individual partecipant data that underlie the results reported in this article will be shared, after deidentification beginnig 3 months and ending 5 years following article publication, with Researchers who provide a methodologically sound proposal. Proposal should be directed to eugeniomaria.mercuri@policlinicogemelli.it; to gain access, data requestors will need to sign a data access agreement.

## Abbreviations

CHOP INTEND: Children’s Hospital of Philadelphia Infant Test of Neuromuscular Disorders
OA: Onasemnogene Abeparvovec
OrSAT: Oral and Swallowing Abilities Tool
PEG: Percutaneous Endoscopic Gastrostomy
SALT: Speech and Language Therapist
SMA: Spinal Muscular Atrophy
SMN: Survival Motor Neuron
