# Supplementary figures and images for: Prognostic factors for tube feeding in type I SMA patients treated with disease-modifying therapies: a cohort study
Source: Eur J Pediatr. 2024 Aug 29;183(11):4735–45. doi: 10.1007/s00431-024-05735-9 (PMC11473555; doi:10.1007/s00431-024-05735-9)

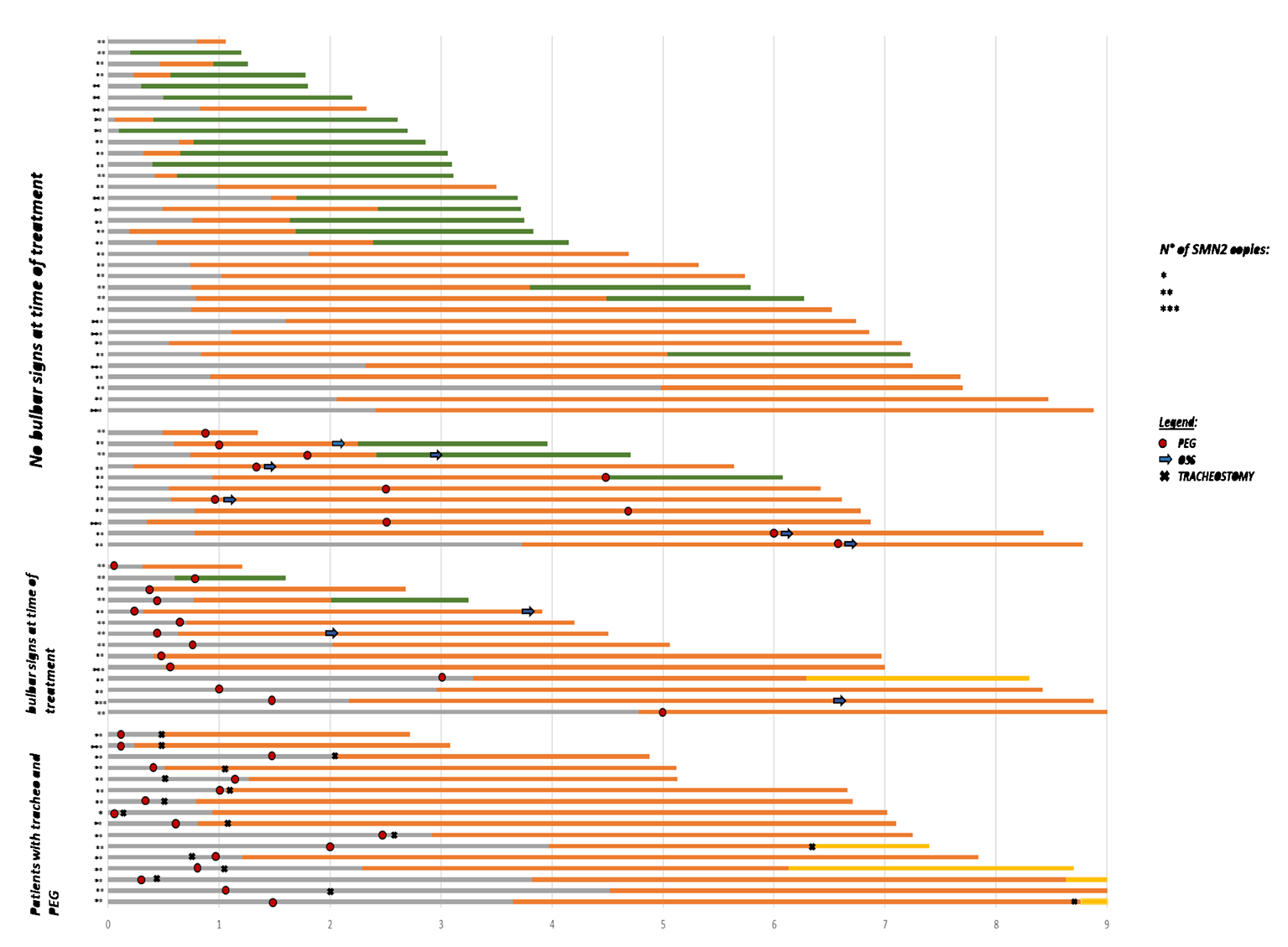

Supplement: Supplementary file 1 — Supplementary file1 Figure 1S. Individual details of the type of treatment and of the age when this was started. Key to the figure. Asterisk: number of SMN2 copies; red circle: insertion of PEG; blue arrow: restarted to eat by mouth; black cross: insertion of tracheostomy. The green lines indicate treatment with onasemnogene abeparvovec, the orange lines indicate treatment with Nusinersen; the yellow lines indicate treatment with Risdiplam and the grey ones indicate the time without any treatment. (PNG 145 kb) [file 431_2024_5735_Fig6_ESM.png]

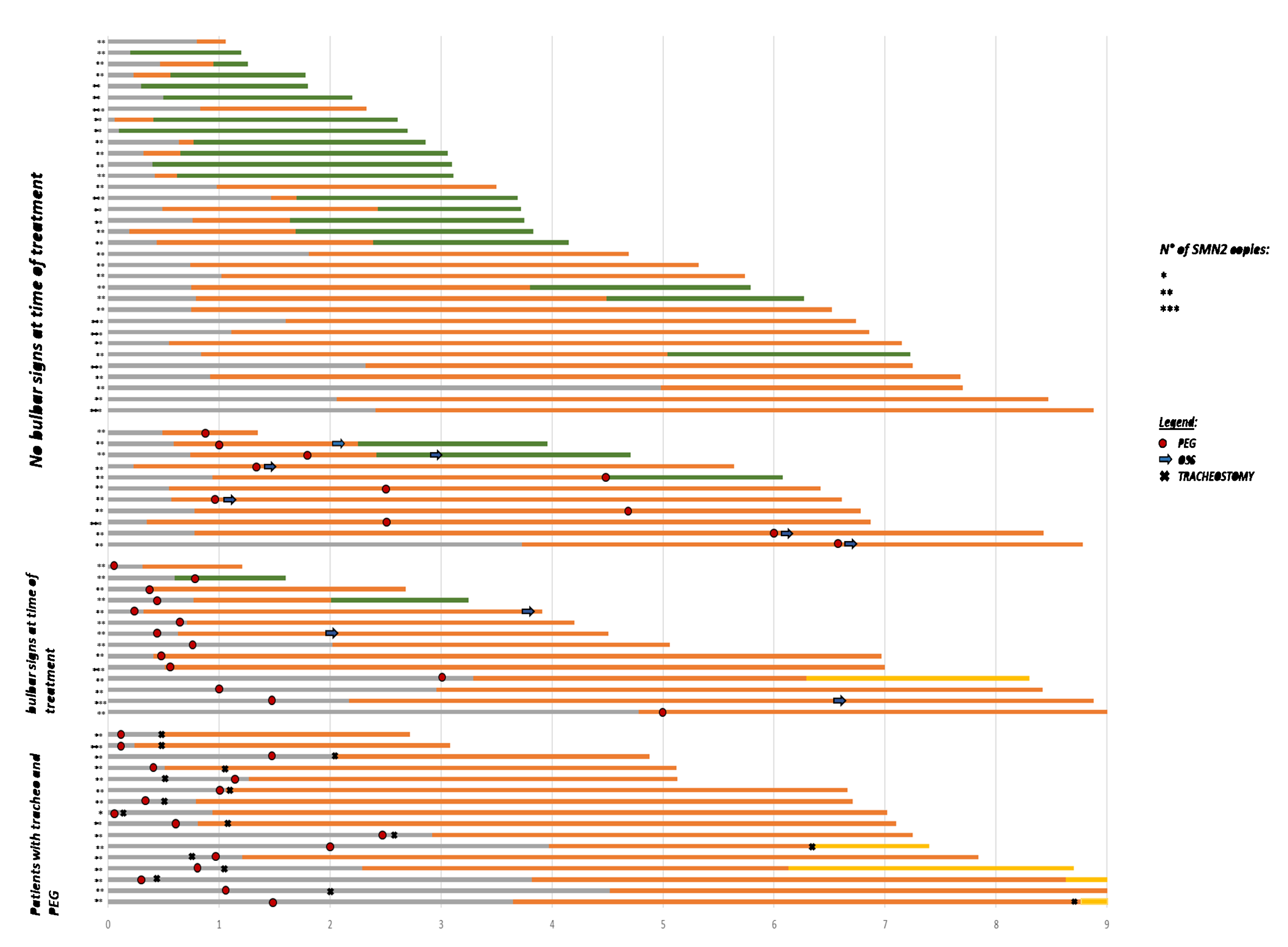

Supplement: Supplementary file 2 — High resolution image (TIF 412 kb) [file 431_2024_5735_MOESM1_ESM.tif]
